# Immunotherapy resistance of lung cancer

**DOI:** 10.20517/cdr.2021.101

**Published:** 2022-02-08

**Authors:** Xin Yu, Chaonan Han, Chunxia Su

**Affiliations:** Department of Oncology, Shanghai Pulmonary Hospital & Thoracic Cancer Institute, Tongji University School of Medicine, Shanghai 200433, China.; **^#^** Authors contributed equally.

**Keywords:** Immunotherapy, resistance mechanisms, response strategies

## Abstract

In recent years, immunotherapy has made remarkable breakthroughs and brought long-term survival benefits to lung cancer patients. However, a high percentage of patients do not respond to immunotherapy or their responses are transient, indicating the existence of immune resistance. Current studies show that the interactions between cancer cells and immune system are continuous and dynamic. A range of cancer cell-autonomous characteristics, tumor microenvironment factors, and host-related influences account for heterogenous responses. Furthermore, with the identification of new targets of immunotherapy and the development of immune-based combinations, we propose the response strategies to overcome resistance.

## INTRODUCTION

**Figure 1:**
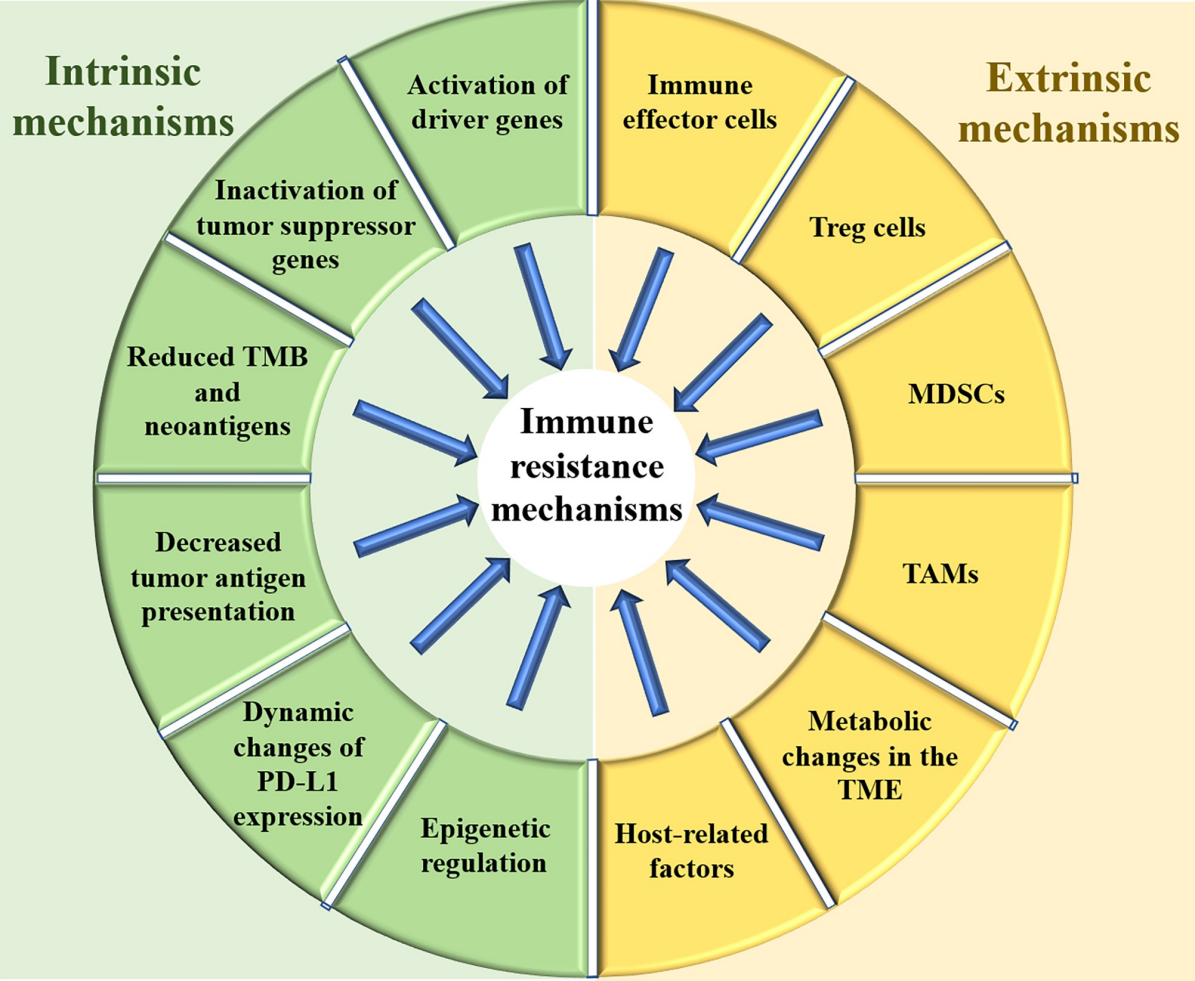
The intrinsic and extrinsic mechanisms and factors of immune resistance. TMB: Tumor mutation burden; PD-L1: programmed cell death ligand 1; MDSCs: myeloid-derived suppressor cells; TAMs: tumor-associated macrophages; TME: tumor microenvironment.

According to the latest cancer report of China, the morbidity and mortality of lung cancer are still the highest among all types of malignancies. Immune checkpoint inhibitors (ICIs) have dramatically changed the treatment landscape for advanced non-small cell lung cancer (NSCLC). In neoadjuvant therapy, ICIs combined with chemotherapy have increased the chances of cure for patients with early-stage NSCLC. In maintenance treatment, durvalumab significantly prolonged the disease-free survival time of NSCLC after concurrent radiotherapy and chemotherapy. The five-year survival rate of patients with advanced NSCLC who received ICI monotherapy increased from 5% to 15%. At the same time, ICIs combined with chemotherapy have also brought a giant breakthrough in the treatment of advanced small cell lung cancer (SCLC). However, the majority of patients treated with ICIs are either non-responders or eventually develop progressive disease^[1,2]^. Therefore, clarifying resistance mechanisms and proposing response strategies for immunotherapy are ongoing challenges that need to be coped with. The tumor is a heterogeneous and dynamic tissue, which continuously evolves in the attempt to overcome structural, metabolic, and immunologic hurdles. Thus, resistance mechanisms and pathways are intertwined. Generally, the resistance mechanism of immunotherapy can be classified into two categories: intrinsic mechanisms and extrinsic mechanisms. Intrinsic mechanisms refer to the genetic, transcriptional or functional profile of the tumor cells themselves, while the extrinsic mechanisms refer to the tumor microenvironment and factors other than the tumor itself. The changes of immunosuppressive cells, immunosuppressive cytokines, coinhibitory receptors, and costimulatory receptors in the tumor microenvironment can destroy the antitumor immune response, mediating resistance to immunotherapy. Host-related factors including physical status, previous comorbidities, distribution of intestinal flora, and use of antibiotics could also affect the efficacy of immunotherapy. This opinion article intends to discuss the currently known mechanisms of immune resistance and potential response strategies in lung cancer. The mechanisms and factors of immune resistance discussed in this opinion are summarized in Figure 1.

## RESISTANCE MECHANISMS OF IMMUNOTHERAPY IN LUNG CANCER

### Intrinsic mechanisms and factors of resistance

The intrinsic factors and mechanisms of resistance mainly refer to the expression or inhibition of specific genes and pathways of tumor cells, such as the activation of driver genes and the inactivation of suppressor genes, low tumor mutation burden (TMB), decreased tumor antigen presentation, and changes in the expression of programmed cell death ligand 1 (PD-L1) of tumor cells.

#### Activation of driver genes

Genomic or epigenetic alternations in tumor cells can mediate immune escape. Several driver genes have been identified in lung cancer, such as epidermal growth factor receptor (EGFR), anaplastic lymphoma kinase (ALK), and Kirsten rat sarcoma virus oncogene homolog (KRAS). They have all been confirmed to be related to the process of immune escape.

EGFR mutation is the most common type of mutation of NSCLC in Asians, with an incidence of 40.3%-64.5%^[3]^. Clinical trials showed that these patients do not respond well to immune checkpoint monotherapy^[4-6]^. There are several hypotheses for the inferior response of immunotherapy in this group of people. First, the immune microenvironment of this population belongs to the immune desert or immune excluded type with poor outcomes of immunotherapy^[7,8]^. After the activation of EGFR pathway, the ligand amphiregulin (AREG) could promote the production of regulatory T (Treg) cells to enhance the immunosuppressive effect through EGFR-glycogen synthase kinase-3β (GSK-3β)-forkhead box P3 (FOXP3) axis^[9]^. Besides, the EGFR signaling pathway can also produce inhibitory cytokines, induce myeloid-derived suppressor cells (MDSC) and tumor-associated macrophages (TAM) proliferation, and inhibit CD8+ T Cell response^[10]^. Second, the signal transducer and activator of the transcription 3 (STAT3) from the downstream of the signal pathway is upregulated, which in turn leads to the decreased expression of major histocompatibility complex I^[11]^. STAT3 also mediates the expression of vascular endothelial growth factor (VEGF), interleukin-6 (IL-6), and interleukin-10 (IL-10) and inhibits the differentiation and maturation of dendritic cells (DC). These changes will ultimately affect the presentation of antigens and the production of new antigens. Third, although most studies have demonstrated that EGFR will upregulate PD-L1, due to the decrease of new antigens and the increase of immunosuppressive cells, patients have not received durable benefits from immune checkpoint monotherapy^[10]^.

Another common gene mutation in lung cancer is ALK fusion. ALK fusion can reduce the production of new antigens and increase the number of immunosuppressive cells through the PI3K-AKT and MEK-ERK pathways, resulting in poor efficacy of immune checkpoint monotherapy^[12,13]^. *KRAS* is another common driver gene in lung cancer. Studies showed that KRAS mutation could increase the expression of PD-L1 to promote immune escape by regulating the stability of the 3'UTR region of PD-L1 mRNA^[14]^. However, KRAS mutations are correlated with an inflammatory tumor microenvironment and tumor immunogenicity, resulting in superior patient response to PD-1/PD-L1 inhibitors. In a single-center retrospective study which included 25 cases of KRAS mutations and 47 wild-type cases, patients were administered with nivolumab ± anti-CTLA-4 antibody. The median overall survival (OS) of the two groups was 18.1 and 8.1 months, respectively (HR = 0.48, *P* = 0.04)^[15]^. Mechanically, KRAS mutations induce the expression of neoantigens and change the expression a series of genes, such as cell cycle regulation, DNA replication, and DNA repair, but they have no obvious effect on activating immunosuppressive cells^[16]^. Several driver mutations of NSCLC and their chances to receive ICI therapy are summarized in Table 1.

**Table 1:** Common driver mutations of NSCLC and their chances to receive ICI therapy

**EGFR mutation**		**ALK mutation**		**KRAS mutation**			**Other mutations**
ICI monotherapy	ICI combination therapy	ICI monotherapy	ICI combination therapy	Single mutation	KP co-mutations	KL co-mutations	MET/RET/ROS1/BRAF/HER2
×	√	×	?	√	√	×	?
Not recommended	Consider using ICIs in some cases	Not recommended	Consider using ICIs in some cases	Probably benefit from ICIs	Probably benefit from ICIs	Not recommended	No large-scale trials and need further research

NSCLC: Non-small cell lung cancer; ICIs: immune checkpoint inhibitors; EGFR: epidermal growth factor receptor; ALK: anaplastic lymphoma kinase; KRAS: Kirsten ratsarcoma viral oncogene homolog; KP: KRAS/TP53; KL: KRAS/STK11; MET: mesenchymal to epithelial transition factor; RET: ret proto-oncogene; ROS1: c-ros oncogene 1; BRAF: v-raf murine sarcoma viral oncogene homolog B; HER2: human epidermal growth factor receptor-2.

#### Inactivation of tumor suppressor genes

The inactivation of tumor suppressor genes, such as *PTEN* and *STK11* genes, can also lead to poor outcomes of immunotherapy for lung cancer patients. In drug-resistant populations, the occurrence of the tumor suppressor PTEN mutations are significantly increased^[17]^. PTEN is a lipid phosphatase that inhibits the activity of the PI3K pathway. Current studies have shown that loss of PTEN expression is the most common way to activate the PI3K pathway in multiple cancers^[18]^. The PI3K pathway plays an indispensable role in tumor growth by regulating tumor proliferation and survival. Another study found a decreased expression of tumor-infiltrating lymphocytes (TILs) in patients with a high deletion rate of *PTEN* gene^[19]^. In addition, PTEN can upregulate the expression of VEGF and prevent tumor immune escape mediated by lymphocyte infiltration. *STK11*/*LKB1* is another tumor suppressor gene. About 8%-39% of patients with NSCLC have *STK11* gene mutation, which has the function of negatively regulating the rapamycin signal pathway. *STK11* gene mutations are often accompanied by a decreased infiltration of cytotoxic CD8+ T lymphocytes, leading to the “cold” immune microenvironment^[20]^. Co-mutations of KRAS and STK11 (KL) or TP53 (KP) could also affect the efficacy of ICI therapy. Skoulidis *et al*.^[21]^ reported that patients with KRAS mutations and STK11/LKB1 alterations had reduced efficacy to immunotherapy in a cohort of 165 patients diagnosed with KRAS-mutated lung adenocarcinoma who had received PD-1/PD-L1 inhibitors treatment. In a mice model of KRAS mutation, the deletion of *STK11* gene promotes the resistance of PD-1 inhibitors monotherapy. Thus, KL tumors show resistance to PD-1 inhibitors, while KP tumors are more sensitive. Besides, the mutation rate of *SMARCA4* gene in NSCLC is about 10%, which is responsible for encoding the catalytic unit of the SWI/SNF chromatin remodeling complex. A study included 2690 NSCLC patients, of whom 211 patients carried SMARCA4 mutations^[22]^. Further analyses revealed that KRAS and SMARCA4 co-mutations are the most prevalent type. Among the NSCLC patients harboring KRAS mutations, patients with the co-occurrence of SMARCA4 mutation had a reduced objective response rate (ORR) (0.0% *vs*. 23.5%, *P* = 0.02), progression-free survival (PFS) (1.7 months *vs*. 4.1 months, *P* < 0.001), and median OS (4.0 months *vs*. 14.9 months, *P* < 0.001). In summary, mutations of driver genes and tumor suppressor genes can lead to changes in tumor neoantigens and can also alter the composition of the immune microenvironment, which is reflected in the heterogeneous efficacy of immunotherapy. Thus, it is necessary to interpret its impact on the efficacy of immunotherapy and the predictive value of drug resistance in clinical practice.

#### Reduced TMB and neoantigens

TMB is the number of substitution, insertion, and deletion mutations per megabase in the exon coding region of the evaluated gene. Yarchoan *et al*.^[23]^ analyzed the TMB of 27 tumor types and found a superior ORR of immune checkpoint monotherapy for tumor types with high TMB. NSCLC and melanoma have relatively higher mutation loads and immunogenicity, and they are more sensitive to ICI therapy. On the contrary, pancreatic cancer, prostate cancer, and thyroid carcinoma have lower mutation loads and exhibit lower immune responses^[23-25]^. Tumor neoantigens refer to antigens that are not expressed in normal tissues but only exist in tumor tissues, which are usually highly immunogenic. Several studies have shown that increased TMB, DNA mismatch repair gene deletion, and high genomic microsatellite instability can increase tumor antigen expression and improve immune efficacy^[24,26]^. Anagnostou *et al*.^[27]^ found that the neoantigen load of NSCLC patients treated with pembrolizumab with sustained clinical benefit was significantly higher than that of patients with non-sustained benefit. The study also found that 7-18 mutation-related neoantigens that can produce anti-cancer responses were lost and replaced by more complicated new mutations before drug resistance. The proportion of new mutations involved in encoding tumor antigens had decreased (8% *vs*. 19%) and the clonality of T cell receptors (TCR) was changed, leading to the acquired drug resistance.

#### Decreased tumor antigen presentation

The major histocompatibility complex (MHC) is a key molecule involved in the immune response, participating in the processing and presentation of antigens, thereby activating T cells and mediating the immune response. β2-microglobulin (β2-GM) is one of the important proteins that compose the heavy chain of MHC class I molecules, which is responsible for folding and transporting MHC class I molecules to the cell surface^[28]^. Studies confirmed that *B2M* gene mutations can lead to the loss of MHC class I molecules on the cell surface, which in turn promotes the recognition dysfunction of CD8+ T cells and induces immune resistance in NSCLC^[29]^. In addition, studies found that defective expression of MHC class II molecules in tumor cells and infiltrating lymphocytes cannot effectively activate T helper cells. The low antigen presentation reduces the immunogenicity of lung cancer, while weak immunogenic tumors can escape the surveillance of the immune system and promote immunotherapy resistance in lung cancer^[30]^.

#### Dynamic changes of PD-L1 expression

Tumor cells with high expression of PD-L1 can also mediate immune escape. PD-L1 could transmit negative signals and induce T cell apoptosis or dysfunction when combined with its receptor, thereby evading the immune system. In patients with acquired resistance, studies have found that interferon-γ (IFN-γ) secreted by both TILs and tumor antigen-specific T cells can mediate the upregulation of PD-L1 expression after tumor antigen recognition. In addition, there are many ways for tumors to upregulate the expression of PD-L1. For example, the currently known PTEN deletion^[18]^, PI3K, or AKT mutations^[31]^ can induce the expression of PD-L1 in tumor cells to escape from immune system. However, clinical trials including KEYNOTE-024 and KEYNOTE-208 have demonstrated that the high PD-L1 expression of tumor cells were more sensitive to immunotherapy^[32,33]^. Chinese Society of Clinical Oncology guidelines and The National Comprehensive Cancer Network guidelines also indicate that the expression level of PD-L1 can be used as one of the predictors of the efficacy of immunotherapy. These studies suggest that the functions of PD-L1 may be diverse, rather than simply categorized as “promoting” or “inhibiting”. A recent study confirmed that deacetylation-dependent nuclear translocation of PD-L1 promotes immune escape and upregulates the immune checkpoint genes such as PD-L2 and VISTA of tumor cells, thereby mediating resistance to PD-1/PD-L1 inhibitors^[34]^. In addition, nuclear PD-L1 can also induce immune response-related gene expression, including type I and type II IFN signaling pathways, nuclear factor kappa-B (NF-κB) signaling pathways, and antigen presentation pathways. These results indicate the dual role of PD-L1, which participates in both drug resistance and immune response pathways.

#### Epigenetic regulation

Epigenetics is the study of heritable changes in gene expression caused by mechanisms other than changes in the nucleotide sequence of the gene, including DNA methylation, histone modification, nucleosome remodeling, and non-coding RNA expression. Epigenetic changes can affect the expression of immune checkpoints, disrupt the process of antigen presentation, and inhibit the migration of T cells to the tumor microenvironment and the activation of T cells^[35]^. In addition, miRNA is also involved in the above process. Studies have shown that miR-214, miR-126, and miR-568 could promote the development and function of Treg cells and lead to T cell exhaustion^[35]^.

### Extrinsic mechanisms and factors of resistance

The extrinsic mechanisms and factors of resistance mainly refer to the tumor microenvironment (TME). The TME is the internal environment for the emergence and growth of tumor cells. Immunologists have tried to find ways to positively affect the immune response, such as the usage of cytokines, adoptive immunotherapy, and other treatments, but the results are not satisfactory. Recently, researchers are focusing on the balance between the “positive factors”, such as immune effector cells, and “negative factors”, such as Treg cells and MDSC in TME to enhance the efficacy of immunotherapy.

#### Immune effector cells

The lower infiltration and exhaustion of immune effector cells lead to immune suppression and mediate immune escape. Based on the distribution of CD8+ T cells, the immune phenotypes are divided into three types: immune inflamed, immune excluded, and immune desert. In a phase II clinical trial of durvalumab combined with olaparib in SCLC, 14 evaluable patients were included, of whom nine were immune excluded, three were immune-inflamed, and two were immune desert type. They study found that immunotherapy may play a role in the TME infiltrated by CD8+ T cells^[36]^. Guo *et al*.^[37]^ analyzed the single-cell sequencing of 12,346 T cells in the peripheral blood, cancer tissue, and adjacent tissues from 14 patients with NSCLC, and they found that infiltrating T cells were mainly comprised of three subgroups. In addition to tumor-infiltrating CD8+ T cells undergoing exhaustion, two other clusters of cells exhibited states preceding exhaustion, and a high ratio of “pre-exhausted” to exhausted T cells was associated with better prognosis of lung adenocarcinoma. NK cells are not restricted by MHC or antibodies and can directly release perforin and TNF to exert their immune functions. The dysfunction of NK cells could mediate immune escape through such mechanisms as overexpression of inhibitory receptors and impaired production of cytokines. Trefny *et al*.^[38]^ analyzed 35 patients with NSCLC who were treated with nivolumab and found a correlation between immunoglobulin-like receptor gene *KIR3DS1* expressed by NK cells and therapeutic efficacy. Mutations of *KIR3DS1* gene could lead to resistance to immunotherapy. In addition, several studies have revealed the relationship between the immune response of B cells and tertiary lymphoid structures (TLS). Compared with non-responders, responders have significantly higher B cell-related gene expression levels before ICI treatment. The density of CD20-positive B cells and TLS in the tissues and the ratio of TLS to tumor area in patients who respond to immunotherapy were significantly higher^[39-41]^. In general, these studies have shown the role of immune effector cells in regulating anti-tumor immunity and immunotherapy efficacy.

#### Treg cells

Treg cells are a type of T cell group with immunosuppressive function, which can inhibit the activation and proliferation of CD4+ and CD8+ T cells and the functions of naive and memory T cells. Treg cells play an indispensable role in maintaining the balance of autoimmunity and can effectively weaken the immunity of autoantigens. However, they are also “utilized” by tumors cells that could express autoantigens to avoid immune surveillance^[42]^. Elevated Treg levels were found in lung, breast, and pancreatic cancers^[43]^, while the Treg levels were significantly reduced after surgical removal of the tumor^[44]^. Animal experiments^[45]^ have proved that the depletion of CD25+ Treg cells could effectively improve the anti-tumor immune response of mice. Thus, it is believed that the elimination of Treg cells should be an essential part of the treatment. Several chemotherapeutic drugs are known to interfere with the function of Treg cells non-specifically, including cyclophosphamide, gemcitabine, methotrexate, and thalidomide^[46]^. Drugs for Tregs targeting CD25 and CTLA-4 and eliminating tumor-related Treg cells include the well-known CTLA-4 antibodies ipilimumab and tremelimumab. In addition, the anti-CD25 antibody daclizumab^[47]^, the anti-OX40 (CD134) monoclonal antibody^[48]^, and the chemokine receptor 8^[49]^ are in phase I clinical trials, which bring hopes for the treatment of cancer patients.

#### MDSCs

MDSC is a type of cell population with immunosuppressive function, which can mediate immune escape and immune tolerance through a variety of ways^[50]^. In addition to secreting molecules such as TGF-β and IL-10 that directly inhibit T effector cells, MDSCs can also induce the proliferation of other immunosuppressive cells. Studies have shown that MDSCs could promote the production of FOXP3+ Treg cells by secreting IFN-γ and IL-10^[51,52]^. MDSCs can also exert immunosuppressive effects by blocking lymphocyte homing and regulating enzymes required for adenosine metabolism. Kim *et al*.^[53]^ found that the MDSC level of tumor-bearing mice that are resistant to ICIs increased by 5-7 times compared with non-tumor-bearing mice. They also found that a high level of PI3Kγ in MDSCs can promote the production of inflammatory mediators and immunosuppressive factors. When combining PI3K inhibitors with PD-1/CTLA-4 inhibitors, the MDSC level of tumor-bearing mice decreased significantly and was comparable to the control group. De Henau *et al*.^[54]^ verified that PI3Kγ inhibitors could remodel the immune microenvironment and prevent tumor growth. High expression of indoleamine 2,3-dioxygenase (IDO) was found on MDSCs in immune-resistant lung cancers. Previous studies showed that the expression level of IDO was positively correlated with clinical stages^[55]^. The application of IDO inhibitor INCB023843 downregulates the expression of IDO and increases the infiltration of CD8+ T cells, thereby reactivating the T cell anti-tumor response^[56]^. These studies reveal the potential value of suppressing MDSCs and its combination with immune checkpoint inhibitors.

#### TAMs

Circulating monocytes and macrophages are recruited into the tumor site and change the tumor microenvironment in the process of tumor progression. The phenotypes of macrophages could be transformed with the changes of signals produced by tumor and stromal cells. Macrophages could be divided into two categories based on their functions. M1 type macrophages are related to inflammatory response, pathogen elimination, and anti-tumor immunity, while M2 type macrophages have tumor-promoting properties. TAMs are very similar to M2 type macrophages^[57]^. Studies of multiple tumor types including breast cancer^[58]^, ovarian cancer^[59]^, and NSCLC^[60]^ have shown that the aggregation of macrophages is positively correlated with CCL2 levels. In addition to CCL2, other chemokines such as CCL3, CCL4, CCL5, and cytokines are also involved in the recruitment of macrophages^[61,62]^. TAMs inhibit the function of T cells by weakening the ability of antigen presentation and releasing immunosuppressive factors such as IL-10 and TGF-β. Studies have shown that the inhibition of the recruitment of macrophages through the regulation of chemotactic agents is effective. For example, bindarit is used to inhibit CCL2^[63]^, and monoclonal antibodies that block VEGFR2 reduce macrophage infiltration and tumor growth^[64]^. CSF-1R blockade can also reduce the number of TAMs and activate T cells in TME^[65]^. These preclinical trials have provided a basis for reversing the drug resistance raised by TAMs.

#### Metabolic changes in the TME

Metabolic changes in the TME can reduce immune effects by producing immunosuppressive metabolites to inhibit immune cell infiltration^[66]^. For example, some tumors use glutamine as an energy source, and the metabolically decomposed ammonia can activate autophagy in neighboring immune cells. Arginine metabolism plays an important role in the activation of T cells and the regulation of immune response. The immunoregulatory cells expressing arginase 1 (ARG1) in TME degrade arginine and restrict its use by T cells. Therefore, inhibition of arginase in TME can enhance the efficacy of ICIs. A clinical trial of the combination of ARG1 inhibitor INCB001158 and nivolumab is ongoing. Besides, the rate of fatty acid synthesis is increased to produce cell membrane phospholipids and signal molecules in tumor cells, and targeting these metabolic pathways is a promising way to enhance anti-tumor immunity. In recent years, many studies have focused on the metabolic process of the TME, providing new perspectives for overcoming immune resistance.

#### Host-related factors

The immune system function degenerates with age, and the number and function of antigen-presenting cells and lymphocytes are also decreased^[67]^. A study found that elderly patients with melanoma responded better to immunotherapy than younger people^[68]^. A meta-analysis including 11,351 cases with advanced or metastatic cancer found that male patients had superior efficacy of immunotherapy^[69]^. Compared with the control group, the overall survival HR of male and female patients treated with ICIs was 0.72 and 0.86, respectively (*P* = 0.0019). In addition, studies have suggested that weight can also affect the efficacy of immunotherapy. A study conducted a pooled analysis of four studies of OAK, POPLAR, BIRCH, and FIR, reporting a correlation between the overall survival of atezolizumab in patients with advanced NSCLC and body mass index^[70]^. The results show that obesity was associated with a significant increase in the OS of patients receiving PD-L1 inhibitors treatment, and the risk of death in obese patients was reduced by 64% (HR = 0.36, 95%CI: 0.21-0.62). Smoking is also a crucial factor. The average frequency of gene mutations in smokers is more than 10 times higher than that of non-smokers^[71]^. A recent study reported that benzopyrene in cigarettes can induce an increase in PD-L1 level, which further explains why smoking patients respond better to immunotherapy^[72]^. In addition, studies have shown that gut microbiota have a significant impact on the efficacy of immunotherapy^[73]^. There is evidence of the importance of the intestinal microbiota in the response to chemotherapy and immunotherapy and how their alteration and the concomitant use of antibiotics inhibit the benefit of ICIs in advanced cancer, decreasing OS and PFS in NSCLC. With respect to microbiota composition, the relative abundance of *Akkermansia muciniphila *appears to significantly affect the clinical response to anti-PD-1/PD-L1 therapy in NSCLC and renal cell carcinoma^[74]^. Another study demonstrated that a greater diversity of the gut microbiome is related to favorable responses to ICI therapy in NSCLC^[75]^. Among the different immune cells, the microbiota have been shown to be associated with the development of effector cells of the immune system, such as Th1, Th2, Th17, and Treg cells^[76-78]^. However, in the case of SCLC, there is little evidence that supports the role of the microbiome as an immune resistance mechanism. Thus, host-related factors could also affect the efficacy of immunotherapy.

## IMMUNOTHERAPY-BASED COMBINATIONS TO COPE WITH RESISTANCE

There are no standard treatments after resistance of immunotherapy. At present, the most promising and effective treatment methods are immunotherapy combinations that aim to convert “cold” tumors with lower immune response into “hot” tumors with better response. A variety of combined treatment strategies, such as immunotherapy combined with targeted therapy, chemotherapy, and radiotherapy can modulate the immune response at different stages and overcome drug resistance. Clinical trials of immunotherapy-based combinations related to lung cancer patients are summarized in Table 2.

**Table 2:** Clinical trials of immunotherapy-based combinations related to lung cancer patients

**Immunotherapy-based combinations**	**Combination strategies**	**Drugs**	**Clinical trials**
**Dual immunotherapy**	Anti-PD-1 + Anti-CTLA-4	Nivolumab + Ipilimumab	Checkmate 227 (NCT02477826)
	Anti-PD-1 + Anti-LAG-3	Nivolumab + MK-4280	Keynote 495 (NCT03516981)
		Nivolumab + Relatimab	NCT03607890
		Pembrolizumab + IMP321	NCT03625323
	Anti-PD-1/PD-L1 + Anti-TIGIT	Atezolizumab + Tiragolumab	CITYSCAPE (NCT03563716)
		Pembrolizumab + MK-7684	NCT02964013
	Anti-PD-L1 + Anti-TIM-3	LY3300054 + LY3321367	NCT03099109
**ICIs combined with CT**	Anti-PD-1/PD-L1 + CT	Pembrolizumab + Pemetrexed/Platinum	Keynote 189 (NCT02578680)
			Keynote 021 (NCT02039674)
		Atezolizumab + Paclitaxel/Carboplatin	IMpower 130 (NCT02367781)
			IMpower 131 (NCT02266949)
		Atezolizumab + Etoposide/Carboplatin	IMpower 133 (NCT02763579)
**ICIs combined with targeted therapy**	Anti-PD-L1 + Anti-VEGF + CT	Atezolizumab + Bevacizumab + Paclitaxel/Carboplatin	IMpower 150 (NCT02366143)
	Anti-PD-L1 + Anti-RTK	Nivolumab + Sitravatinib	NCT02954991
	Anti-PD-L1 + Multitarget inhibitor	Atezolizumab + Cabozantinib	COSMIC-021 (NCT03170960)
**ICIs combined with RT**	Durvalumab after concurrent RT + CT		PACIFIC (NCT02125461)
	Pembrolizumab + SBRT		PEMBRO-RT (NCT02492568)
**Individualized immunotherapy**	Anti-PD-1 + Vaccine	Nivolumab + NEO-PV-01	NCT02897765
	Anti-PD-1 + MDM2 inhibitor	Pembrolizumab + APG-115	NCT03611868

ICIs: Immune checkpoint inhibitors; PD-1: programmed cell death 1; PD-L1: programmed cell death ligand 1; CTLA-4: cytotoxic T lymphocyte-associated antigen-4; LAG-3: lymphocyte activation gene-3; TIGIT: T cell immunoglobulin and ITIM domains; TIM-3: T cell immunoglobulin-3; CT: chemotherapy; RTK: receptor tyrosine kinases; RT: radiotherapy; SBRT: stereotactic body radiation therapy; MDM2: mouse double minute 2 homolog.

### Dual immunotherapy

Anti-tumor immune response involves antigen recognition, presentation, and activation of immune cells. Currently, many studies focus on the immune checkpoint inhibitors targeting PD-1, PD-L1, lymphocyte activating gene 3 (LAG-3), T cell immunoglobulin domain and mucin domain-3 (TIM-3), and T cell immunoglobulin and ITIM domain protein (TIGIT). The combined application of these immune checkpoint inhibitors could exert synergistic effects. The most common strategy is PD-1 inhibitor combined with anti-CTLA-4 antibody. Both CheckMate 227 and CheckMate 032 studies have confirmed that the efficacy of dual-immunotherapy is better than that of single-agent therapy^[79,80]^. LAG-3 is expressed on the activated CD4+, CD8+ T cells, NK cells, and Treg cells, which can bind to its ligand fibrinogen-like protein-1 (FGL1) to inhibit T cell function. The trial of LAG-3 antibody MK-4280 combined with pembrolizumab in advanced NSCLC (KEYNOTE-495/NCT03516981) recruited 33 patients with solid tumors who have failed previous treatments. The DCR of the monotherapy group and the combination treatment group was 17% and 40%, respectively. TIGIT is a specific negative regulator of CD226 costimulatory receptor and plays an important role in immunosuppression. The CITYSCAPE study enrolled 135 patients with PD-L1-positive treatment-naïve NSCLC who were randomly assigned to TIGIT antibody tiragolumab combined with atezolizumab (TA group) or placebo combined with atezolizumab (PA group). The results show that, in the intention-to-treat population, the ORR and PFS of the TA group was increased compared with the PA group (31.3% *vs*. 16.2%; 5.4 months *vs*. 3.6 months), and the risk of disease progression was reduced by 43%^[81]^. In addition, the phase II clinical trial of relatimab combined with nivolumab in the treatment of solid tumors (NCT03607890) and the phase II clinical trial of IMP321 combined with pembrolizumab in the treatment of patients with advanced NSCLC (NCT03625323) are both underway. The phase I clinical trial of pembrolizumab combined with TIGIT antibody in the treatment of patients with metastatic solid tumors (NCT02964013) and the phase I trial of TIM-3 inhibitor combined with PD-L1 antibody in the treatment of advanced relapsed/refractory solid tumors (NCT03099109) are ongoing.

### Immunotherapy combined with chemotherapy

Immunotherapy combined with chemotherapy can not only increase the cross-presentation of antigens by DCs^[82]^ but also eliminate the immunosuppressive components of the TME^[83]^, such as Treg cells, MDSCs, and immunosuppressive cytokines. The KEYNOTE-189 study showed that the efficacy of pembrolizumab combined with chemotherapy in patients with advanced NSCLC in first line is superior to chemotherapy alone^[84]^. The OS of the two groups was 22.0 and 10.7 months and the PFS was 9.0 and 4.9 months, respectively. In addition, the CheckMate 012, KEYNOTE-021, IMpower130, and IMpower131 studies have shown that immunotherapy combined with chemotherapy has a greater survival benefit than chemotherapy along despite using different checkpoint inhibitors. Based on these studies, the U.S. Food and Drug Administration has approved nivolumab and pembrolizumab combined with platinum-based chemotherapy for EGFR/ALK wild-type NSCLC. Besides, results from the double-blind, randomized phase III study IMpower133 show that, compared with chemotherapy alone, atezolizumab combined with chemotherapy in the first-line treatment of extensive-stage SCLC achieved OS benefit, which represents a breakthrough in the treatment of SCLC^[85]^.

### Immunotherapy combined with targeted therapy

Targeted therapy can increase tumor antigens and play a synergistic effect with immunotherapy in multiple aspects. Anti-angiogenic agents can promote the normalization of blood vessels, enhance immune effector cells to infiltrate the tumor, and reduce the activity of immunosuppressive cells. The IMpower150 study evaluated atezolizumab combined with bevacizumab and chemotherapy in the first-line treatment of non-squamous NSCLC patients, including EGFR and ALK mutant populations, and the results confirm that patients in ABCP group have significant survival benefits^[86]^. Cohort 7 of the COSMIC-021 study included 30 advanced NSCLC patients with negative driver genes who had previously been treated with immune checkpoint inhibitors. The results show that the ORR was 23%, DCR was 83%, and the median duration of response was 5.6 months^[87]^. A phase I randomized clinical study explored the efficacy of nivolumab combined with erlotinib in the treatment of patients with EGFR-mutant advanced NSCLC. Twenty EGFR-TKI resistant cases and one EGFR-naive case were included. The results show that the median PFS was 5.1 months^[88]^. In addition, the combination of novel targeted agents with immunotherapy has also shown great prospects. Nivolumab combined with the small molecule inhibitor sitravatinib in the treatment of patients with advanced non-squamous NSCLC who are resistant to immunotherapy obtained an ORR of 32.14%, a median PFS of 6.8 months, and a median OS of 15.1 months^[89]^. Pembrolizumab combined with JAK1 inhibitor itacitinib in the treatment of NSCLC patients with PD-L1 expression > 50% reported an ORR of 66.7%.

### Immunotherapy combined with radiotherapy

The PACIFIC study included 713 patients with locally advanced unresectable NSCLC. The results show that the median PFS of the durvalumab treatment group after concurrent radiotherapy and chemotherapy was 17.2 months, and the median OS was also significantly improved compared with control group^[90]^. The PEMBRO-RT study explored the efficacy of radiotherapy and concurrent immunotherapy. Patients were treated with pembrolizumab seven days after receiving stereotactic body radiotherapy, while the control group only used immunotherapy. The ORR of the two groups was 36% and 18% and the median OS was 15.9 and 7.6 months, respectively^[91]^. In addition, a retrospective study included 26 NSCLC patients with acquired resistance of immunotherapy. In total, 15 patients were administered with local treatment without systemic treatment, and 11 of them continued to receive immunotherapy after local treatment. The two-year survival rate was 92%, and the median OS has not yet been reached^[92]^. Similarly, a retrospective study including 189 patients with acquired resistance showed that local treatment can significantly improve survival benefits^[93]^.

Other combination strategies are also being explored in immunotherapy-resistant populations. The VARGADO study included 57 patients with advanced NSCLC after progression with second-line immunotherapy. Patients were treated with the VEGF inhibitor nintedanib combined with docetaxel. The results show that ORR and DCR are 50% and 85%, respectively. The median PFS was 6.5 months and the median OS was 12.4 months^[94]^. Although the ideas for overcoming immunotherapy resistance are various, there exist some problems that cannot be ignored while conducting clinical trials. Firstly, adequate pre-clinical evaluation should be carried out when the clinical trial is designed, and the adverse effects should be monitored. For example, in the PACIFIC study, the incidence of radiation pneumonitis in the immunotherapy group was increased (33.9% *vs*. 24.8%), and 15.4% of patients discontinued treatment due to adverse events. In the TATTON study, the incidence of interstitial pneumonitis in NSCLC patients receiving durvalumab combined with osimertinib was significantly increased; thus, the treatment group was promptly interrupted in the corresponding phase III clinical trial (CAURAL study). Novel treatment strategies such as immunotherapy combined with molecular inhibitors need to further consider the potential safety profiles raised by the superposition of different drugs. Secondly, the rationality of the combination strategy is supposed to be evaluated. The selected treatment drugs and immune checkpoint inhibitors should have synergistic effects in the immune feedback loop.

### Individualized immunotherapy

Given the characteristics of the tumor immune microenvironment, developing drugs for tumor cell neoantigen production and presentation, T cell activation, and immunosuppressive microenvironment, such as oncolytic viruses, tumor vaccines, and adoptive immune cell therapy (TIL, CAR-T, TCR-T, CAR-NK, *etc*.), which are based on the characteristics of individualized immune microenvironments represent promising directions for overcoming immunotherapy resistance. The NT-001 study is a phase Ib clinical trial that intends to explore the efficacy of PD-1 inhibitors combined with vaccines for advanced or metastatic melanoma, smoking-related NSCLC, and bladder cancer. In total, 82 patients were enrolled. ORR for metastatic NSCLC patients reached 45.5%. MDM2 (mouse double minute 2 homolog) is one of the most important inhibitors of p53; it degrades the p53 protein and weakens its tumor suppressor effect. APG-115 is a second-generation MDM2 inhibitor that can block the MDM2-p53 interaction, thereby restoring the transcriptional regulatory function of the p53 protein, promoting cell apoptosis, and restoring tumor suppressor activity. The phase Ib clinical trial results of APG-115 combined with pembrolizumab in the treatment of metastatic melanoma or advanced solid tumors confirm that the combination of the two drugs is well tolerated, with an ORR of 16.7% and a DCR of 55.5%. With the continuous in-depth study of immunotherapy mechanisms, individualized immunotherapy targets may be a new strategy with good development prospects.

## CONCLUSIONS AND FUTURE DIRECTIONS

Compared with traditional chemotherapy and targeted therapy, immunotherapy has its unique advantages and brings a new light to lung cancer treatment. With the clinical application of immunotherapy, anti-PD-1/PD-L1 monotherapy resistance has become an unavoidable phenomenon. The ongoing research on the mechanisms of immune resistance provides new ideas for the selection of patient populations and response strategies for reversing immune resistance. Immunotherapy-based combinations are becoming one of the most promising strategies that convert non-responders to responders. In the future, studies on biomarker identifications are required to predict the efficacy and prognosis of immunotherapy. Further exploration is needed to predict immune resistance and the timing of restarting immunotherapy.

## DECLARATIONS

### Copyright

© The Author(s) 2022.
